# Development and Disease-Dependent Dynamics of Spermatogonial Subpopulations in Human Testicular Tissues

**DOI:** 10.3390/jcm9010224

**Published:** 2020-01-14

**Authors:** Joana M. D. Portela, Laura Heckmann, Joachim Wistuba, Andrea Sansone, Ans M. M. van Pelt, Sabine Kliesch, Stefan Schlatt, Nina Neuhaus

**Affiliations:** 1 Center of Reproductive Medicine and Andrology, Institute of Reproductive and Regenerative Biology, Albert-Schweitzer-Campus 1, Building D11, 48149 Münster, Germany; j.m.diogoportela@amsterdamumc.nl (J.M.D.P.); Laura.Heckmann@ukmuenster.de (L.H.); Joachim.Wistuba@ukmuenster.de (J.W.); Andrea.Sansone@ukmuenster.de (A.S.); Stefan.Schlatt@ukmuenster.de (S.S.); 2 Center for Reproductive Medicine, Amsterdam Research Institute Reproduction and Development, Amsterdam UMC, University of Amsterdam, Meibergdreef 9, 1105 AZ Amsterdam, The Netherlands; a.m.vanpelt@amsterdamumc.nl; 3 Department of Experimental Medicine, Sapienza University of Rome, 00161 Rome, Italy; 4 Center of Reproductive Medicine and Andrology, Department of Clinical and Surgical Andrology, Albert-Schweitzer-Campus 1, Building D11, 48149 Münster, Germany; kliesch@ukmuenster.de

**Keywords:** human male infertility, immature testis, fertility preservation, spermatogonia, sickle cell disease, MAGEA4, UTF1, PCNA, 5mC

## Abstract

Cancer therapy and conditioning treatments of non-malignant diseases affect spermatogonial function and may lead to male infertility. Data on the molecular properties of spermatogonia and the influence of disease and/or treatment on spermatogonial subpopulations remain limited. Here, we assessed if the density and percentage of spermatogonial subpopulation changes during development (*n* = 13) and due to disease and/or treatment (*n* = 18) in tissues stored in fertility preservation programs, using markers for spermatogonia (MAGEA4), undifferentiated spermatogonia (UTF1), proliferation (PCNA), and global DNA methylation (5mC). Throughout normal prepubertal testicular development, only the density of 5mC-positive spermatogonia significantly increased with age. In comparison, patients affected by disease and/or treatment showed a reduced density of UTF1-, PCNA- and 5mC-positive spermatogonia, whereas the percentage of spermatogonial subpopulations remained unchanged. As an exception, sickle cell disease patients treated with hydroxyurea displayed a reduction in both density and percentage of 5mC- positive spermatogonia. Our results demonstrate that, in general, a reduction in spermatogonial density does not alter the percentages of undifferentiated and proliferating spermatogonia, nor the establishment of global methylation. However, in sickle cell disease patients’, establishment of spermatogonial DNA methylation is impaired, which may be of importance for the potential use of this tissues in fertility preservation programs.

## 1. Introduction

Male reproductive potential depends on the presence and development of the spermatogonial cell population, which forms the basis for spermatogenesis following puberty. Impairment of spermatogonial development before or during puberty may lead to infertility in adult life. Reasons for this include pathological conditions such as cryptorchidism and genetic or endocrine disorders [1,2,3,4,5]. Apart from that, cancer therapy and conditioning treatments of non-malignant diseases such as sickle cell disease and thalassemia, are known to induce testicular damage, affect spermatogonial function and reduce or even deplete germ cells [4,5,6,7,8,9,10]. Consequently, gonadotoxic treatment may lead to temporary or permanent infertility depending on treatment dose and duration, patients’ age and underlying diagnosis [11,12,13].

Cryopreservation of testicular biopsies containing spermatogonia is the current strategy to preserve reproductive potential of prepubertal patients. Although several approaches are investigated, spermatogonia-based fertility restoration methods to derive sperm from stored testicular tissues remain experimental [14]. Descriptive and qualitative information of the heterogeneous prepubertal testicular tissues is pivotal to determine its potential use in fertility restoration strategies. While accumulating evidence on spermatogonial numbers in bio-banked tissues is reported, evaluation of molecular properties of spermatogonia remains limited [4,5,9,10,15].

From birth onwards, spermatogonia in the human testis are located at the basal membrane of seminiferous tubules [16,17,18]. During childhood testicular development, spermatogonial numbers change depending on their rate of proliferation and apoptosis [19,20,21]. Spermatogonial density tends to slightly decrease from birth to 3 years of age, followed by a gradual increase until approximately 7 years. Thereafter, spermatogonial density remains stable until the age of 11 and considerably increases at the onset of puberty [21].

Subpopulations of adult spermatogonia have been distinguished based on protein marker expression profiles [22,23] and, more recently, by single-cell RNA-sequencing approaches [24,25,26,27]. Consistently, most spermatogonia express germline-specific markers such as melanoma-associated antigen 4 (MAGEA4) [23,28,29,30]. Accumulating evidence reveals that the most primitive spermatogonial state exhibits a transcriptomic profile similar to infant spermatogonia [24,27], with an expression of stem cell-specific transcripts such as the undifferentiated embryonic cell transcription factor 1 (UTF1) and a relatively low proliferative activity [22,24,25,27,31,32]. Transition to a differentiated spermatogonial state is dominated by upregulation of proliferation associated-genes and the loss of markers characteristic of earlier spermatogonial states [24,25,26,27].

In contrast to the heterogeneous spermatogonial transcriptome signatures, stable DNA methylation profiles amongst the various adult spermatogonial subpopulations have been reported. They are suggested to allow developmental plasticity within spermatogonial subtypes [24,33] and ensure gamete integrity [34,35,36], as well as correct transmission of epigenetic information to the next generation [37,38]. The timing of establishment of global DNA methylation in spermatogonia during human testicular development is still undescribed. Employing the marmoset monkey as a non-human primate model, global methylation levels of spermatogonia were reported. Immunohistochemical analysis of 5-methylcytosine (5mC) revealed a progressive increase with development. Specifically, neonatal spermatogonia were predominantly unmethylated (27 ± 1.5% of methylated spermatogonia), then methylation of the spermatogonial population rose during prepubertal (39 ± 23%) and pubertal (83 ± 7.9%) stages until nearly all adult spermatogonia (97 ± 1.7%) were methylated [39]. 

In this study, we aimed to analyze spermatogonial marker expression profiles at various testicular developmental stages of patients at risk of germ cell loss. Composition of spermatogonial subpopulations was evaluated by a marker of spermatogonia (MAGEA4), undifferentiated spermatogonia (UTF1) and functional markers of proliferation (proliferating cell nuclear antigen, PCNA) and methylation (5mC). Furthermore, we evaluated the effect of underlying diseases and/or respective treatment on spermatogonial subpopulations.

## 2. Materials and Methods

### 2.1. Ethical Approval

Ethical approval was obtained from the Ethics Committee of the Medical Faculty of Münster, the State Medical Board (No. 2011-520-f-S) and the Dutch National ethical committee (CCMO) and Medical Ethic Committee (METC) (NL27690.000.09 and 2009-132, respectively) for prepubertal samples and from the Ethics Committee of the Medical Faculty of Münster, the State Medical Board (No. 2016-507-f-S) for adult controls. All patients or legal guardians gave written informed consent to the use of testicular tissue for research purposes.

### 2.2. Testicular Tissue Collection

#### 2.2.1. Immature Patients

Immature testicular tissue was collected from 36 patients included in fertility preservation programs at the Centre of Reproductive Medicine and Andrology (Androprotect; Münster, Germany) and the Academic Medical Center (Amsterdam, The Netherlands). Testicular volumes were determined by palpation using a Prader orchidometer [40] and, in most cases, additionally confirmed by ultrasonography (Table 1). Surgery and testis biopsy collection were performed as previously described [5,41]. The majority of the biopsy was cryopreserved for clinical fertility preservation and the remaining part was fixed in (diluted) Bouin’s solution and further processed for histological analysis.

The patient cohort comprised patients diagnosed *inter alia* with cancer, non-malignant hematological disorders including sickle cell disease and thalassemia, and cryptorchidism. Due to the high variability regarding diagnoses and previous treatments, patients were further divided into two subgroups: NT group included untreated cancer patients without any preexisting (congenital) risk factors for impaired testicular function; AT group comprised patients with testicular tissue potentially affected by pathologies known to affect the testicular function (e.g., cryptorchidism), diagnosed with non-malignant (sickle cell disease, thalassemia, and immunodeficiency), malignant hematological disorders (myelodysplastic syndrome, leukemia and lymphoma), and/or patients who received gonadotoxic treatment (chemotherapy or hydroxyurea) prior to tissue retrieval.

#### 2.2.2. Adult Controls

Adult testicular tissue was obtained from patients who underwent orchiectomy as part of prostate cancer treatment [30,42]. All adult controls (*n* = 7; 62–79 years) had normal spermatogenesis and exhibited a Bergmann-Kliesch score between 8 and 9, meaning that 75–94% of tubules contained elongated spermatids [43]. 

### 2.3. Histological and Immunohistochemical Evaluation of Human Testicular Tissue

Fixed testicular tissues were washed with 70% ethanol and routinely embedded in paraffin. Depending to the research institute, tissue sections of 3 or 5 µm were prepared. Immunohistochemical stainings were performed as published previously [44]. Primary antibodies used in this study included melanoma-associated antigen 4 (MAGEA4), undifferentiated embryonic cell transcription factor 1 (UTF1), proliferating cell nuclear antigen (PCNA), and 5-methylcytosine (5mC) (detailed information is given in Table S1). Respective isotype controls and omission of primary antibodies served as negative controls. Sections were incubated with biotinylated secondary antibodies for 1 h at room temperature followed by incubation with streptavidin conjugated with HRP (S5512, Sigma Aldrich, St. Louis, MO, USA) for 45 min. Immunostaining was visualized with 3,3′-diaminobenzidine and hematoxylin was used as counterstain. Stained sections were entirely scanned using a PreciPoint M8 microscope (PreciPoint, Freising, Germany) at 60× objective.

### 2.4. Spermatogonial Quantification

As immunohistochemically stained sections were suboptimal to discriminate gonocytes, A _dark, pale_ and B spermatogonia, we choose to quantify all spermatogonia irrespective of the subtypes. Quantitative analysis was performed to determine the numbers of spermatogonia expressing MAGEA4, UTF1, PCNA, and 5mC. Due to the limited material, analysis per patient was restricted to one full section for each marker. Counting was performed according to a previously published protocol [5]. Briefly, all positive and negative spermatogonia within the section were counted in an investigator-blinded approach. Spermatogonia were identified based on size, shape, and basal location within the seminiferous tubules [45].

For comparable results, spermatogonial density (numbers per mm^3^; *N_nuc,testis_*) were determined using a previously published equation [46].

$$N_{nuc,testis}=\frac{V_{testis}\;*\;V_{v\;nuc,testis}}{V_{N}}$$

In this equation, *V_testis_* represents the volume of the section (area in mm^2^ * 3 or 5, adjusted according to thickness of tissue section in µm), *V_v nuc,testis_* the number of spermatogonia multiplied by the mean nuclear volume of spermatogonia type divided by the volume of the section, and *V_N_* the spermatogonial mean nuclear volume. All counting and measurements were performed using the ViewPoint software (Precipoint GmbH, Freising, Germany). By measurement of 20 spermatogonia, a mean nuclear diameter of 5.89 ± 0.81 µm was determined. The area of evaluated tissue sections (*n* = 31) ranged from 0.17 to 17.37 mm^2^ in NT patients, 0.46 to 11.31 mm^2^ in AT patients, and 7.29 to 27.80 mm^2^ in adult controls. Numbers of spermatogonia ranged from 44 to 4226 in NT patients, 52 to 4344 in AT patients, and from 1010 to 14,267 in adult controls (Table S2). Nuclear volumes of spermatogonia were calculated using the ball volume formula (volume = 4/3 πr^3^).

To circumvent extrapolation of low spermatogonial numbers to density and as spermatogonial density may vary at different positions throughout the testis, particularly in tissue samples with few spermatogonia; we only included samples in which at least 40 spermatogonia could be counted for each marker for further analyses. Additionally, a patient with germ cell neoplasia in situ (AT21) was excluded to avoid incorrect identification of MAGEA4-positive neoplastic cells as spermatogonia (Table 1 and Table S2).

### 2.5. Spermatogonial Labeling Index

Labeling indices for each marker and patient were determined as the ratio between the number of positively labeled spermatogonia and the total number of spermatogonia. The purpose of this index was to determine the percentage of cells that are positive for each marker irrespective of the absolute cell numbers.

### 2.6. Statistical Analyses

Statistical analysis was performed in R (R Core Team, version 3.5.0), using the dplyr package for data cleaning, the ggplot2 package for plot drawing and the base stats package for creation of generalized linear models. Generalized linear models were used to predict response for the different labeling indices based on the patients’ age. Wilcoxon rank-sum test was used to compare labeling indices during testicular development of the AT group as well as patients with cryptorchidism, sickle cell disease or thalassemia with the NT group. Statistical significance was set at *p*-value < 0.05. Adjustment for multiple comparisons was performed with Bonferroni’s Correction; significance was therefore set at *p* = 0.016 (0.05/3). 

## 3. Results

### 3.1. Density of Spermatogonial Subpopulations during Normal Testicular Development

**Figure 1:**
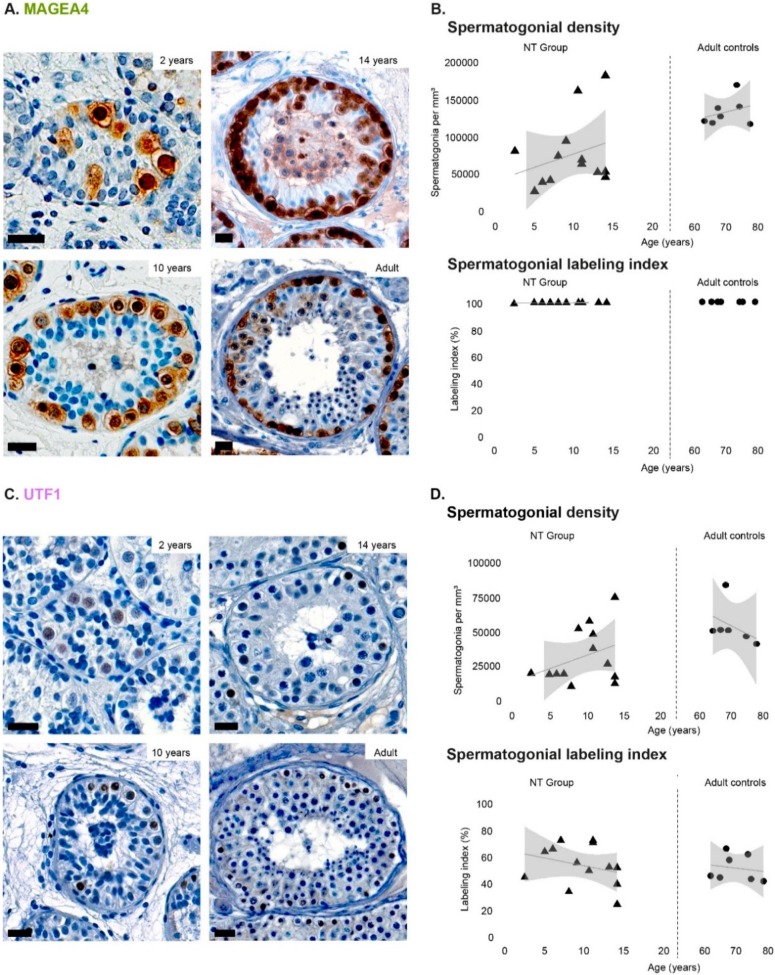
Spermatogonial expression of MAGEA4 and UTF1 throughout age. (**A**) Immunohistochemical identification of MAGEA4-positive (brown) spermatogonia at several testicular developmental stages. Scale bar: 20 µm. (**B**) Density and labeling index (%) of MAGEA4-positive spermatogonia during normal development (NT group) and adulthood. (**C**) Immunohistochemical identification of UTF1-positive (brown) spermatogonia at several testicular developmental stages. Scale bar: 20 µm. (**D**) Density and labeling index (%) of UTF1-positive spermatogonia during normal development (NT group) and adulthood. Individual values, trend line, and 95% confidence interval are represented. Generalized linear models were used to predict the response of labeling index based on the patients’ age.

**Figure 2:**
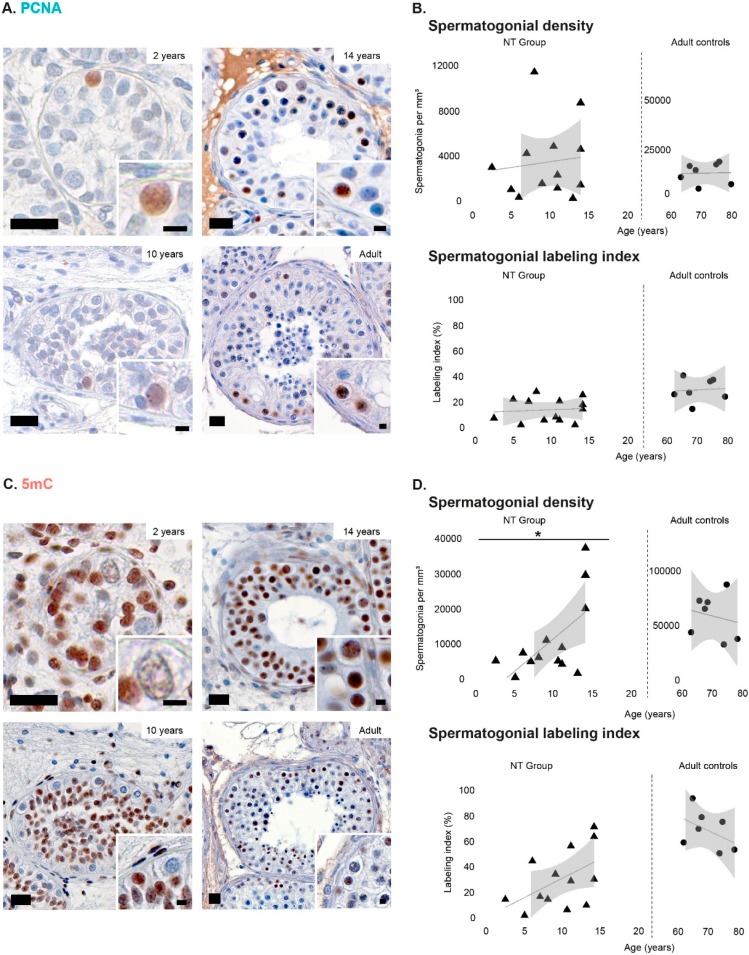
Spermatogonial expression of PCNA and 5mC throughout age. (**A**) Immunohistochemical identification of PCNA-positive (brown) spermatogonia at several testicular developmental stages. Scale bar: 20 µm. (**B**) Density and labeling index (%) of PCNA-positive spermatogonia during normal development (NT group) and adulthood. (**C**) Immunohistochemical identification of 5mC-positive (brown) spermatogonia at several testicular developmental stages. Scale bar: 20 µm. (**D**) Density and labeling index (%) of 5mC-positive spermatogonia during normal development (NT group) and adulthood. Individual values, trend line, and 95% confidence interval are represented. Generalized linear models were used to predict the response of labeling index based on the patients’ age. Statistically significant difference is represented as * *p* < 0.05.

Immature testicular tissue from untreated cancer patients without any evidence of compromised testicular function (NT group) was used to characterize developmental-related changes in spermatogonial marker expression profiles of MAGEA4, UTF1, PCNA, and 5mC. Quantification of spermatogonial density in testicular cross sections (Table S2) from patients included in the NT group revealed a linear tendency of increase throughout testicular development in the density of spermatogonia expressing MAGEA4 (Figure 1A,B), UTF1 (Figure 1C,D), and PCNA (Figure 2A,B), however without statistical significance. In contrast, the density of 5mC-positive spermatogonia (ranging from 214.4 to 36,966.7) significantly increased (*p* < 0.05) with age (Figure 2C,D). In adult control samples, the density of spermatogonia positive for each marker did not change significantly with age (Figure 1B,D and Figure 2B,D).

### 3.2. Spermatogonial Labeling Indices during Normal Testicular Development

To normalize for interindividual variability in spermatogonial density the percentage of marker-positive spermatogonia within the spermatogonial population (labeling index) was determined. Our results reveal that spermatogonial labeling indexes for none of the evaluated markers were significantly influenced by age, neither in the NT patient group nor in adult controls. Briefly, MAGEA4 was expressed by most spermatogonia identified during testicular maturation (NT group) and adulthood (Figure 1B). For UTF1, the spermatogonial labeling index varied between approximately 55% in young patients (less than 5 years old) to ~40–50% during pre-/puberty (5 to 15 years old) and adulthood (Figure 1D). Labeling indices for PCNA-positive spermatogonia were around 10% in young patients, reaching values between 20% and 40% in puberty and adulthood (Figure 2B). Despite a lack of statistical significance, an increasing trend in the 5mC spermatogonial labeling index was observed throughout development. Specifically, 10% of spermatogonia were 5mC-positive in young patients, 55% in post-pubertal patients, and 68% in adult controls (Figure 2D).

**Figure 3:**
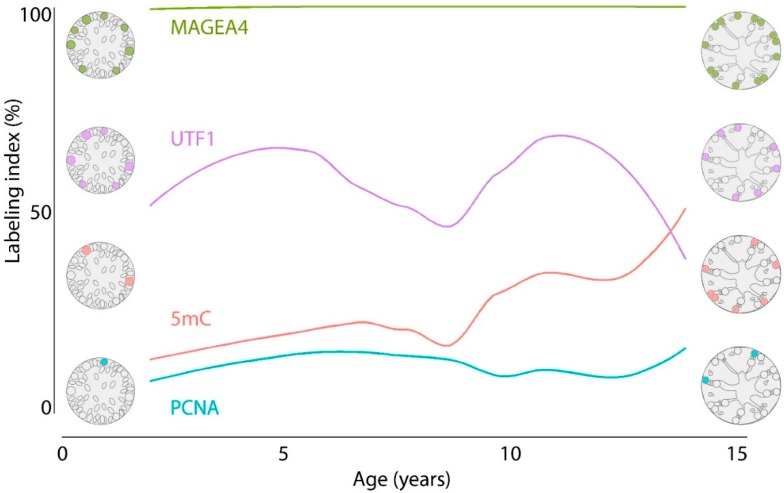
Schematic representation of the dynamic spermatogonial labeling index (%) in testicular tubular cross-sections during normal testicular development. Expression patterns of MAGEA4 (green), UTF1 (purple), 5mC (pink), and PCNA (blue) are the result of smooth interpolation of the data.

For each marker, a dynamic labeling index throughout normal prepubertal testicular development was established by smooth interpolation of the results (Figure 3) and further considered as the reference expression pattern.

### 3.3. Spermatogonial Subpopulations in Testicular Tissues of Patients Affected by Disease or Treatment

The possible effect of underlying diseases and/or respective treatment on MAGEA4, UTF1, PCNA, and 5mC expression profiles in spermatogonia was evaluated by comparison of the AT group with the reference pattern established for normal testicular development (NT group).

**Figure 4:**
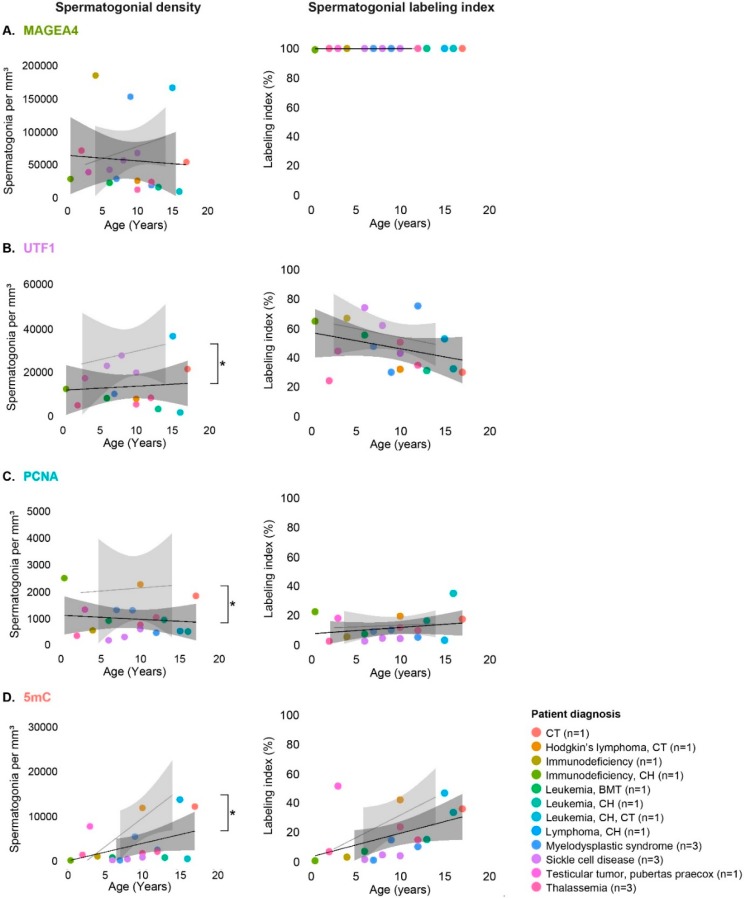
Spermatogonial density and labeling indices (%) of patients with potentially affected tissue by disease and/or treatment (AT group) compared with established values during normal testicular development (NT group). (**A**) MAGEA4, (**B**) UTF1, (**C**) PCNA, and (**D**) 5mC labeling index. Results for each AT patient are represented with individual dots colored according to diagnosis. Previously established reference values throughout age (NT group) are represented as light-gray area (95% confidence interval). Wilcoxon rank-sum test was used to compare labeling indices of the AT group with the reference during testicular development. Statistically significant difference is represented as * *p* < 0.05. Abbreviations: CT—cryptorchid testes; CH—chemotherapy; BMT—bone marrow transplantation.

Compared to the NT group, the density of MAGEA4-positive spermatogonia in tissues from the AT group (Table S2) was reduced with pubertal age, although without significance (Figure 4A). In contrast to this, the density of UTF1- (Figure 4B), PCNA- (Figure 4C), and 5mC-positive (Figure 4D) spermatogonia (Table S2) were significantly lower (*p* < 0.05) throughout age, compared to the reference values (NT group). Particularly the density of 5mC-positive spermatogonia appeared to be identical in younger patients but became significantly reduced with age.

Labeling indices were determined to unravel whether the reduction in the density of spermatogonia expressing MAGEA4, UTF1, PCNA, and 5mC was accompanied by altered ratios of these spermatogonial subpopulations. We found that the percentage of MAGEA4, UTF1, PCNA, and 5mC-positive spermatogonia in the AT patient group was not statistically different from the NT group (Figure 4).

### 3.4. Spermatogonial Marker Expression in Patients with Cryptorchidism, Sickle Cell Disease and Thalassemia

As the AT group comprised patients with diverse diagnoses, we have further analyzed the most prominent pathologies including cryptorchidism (CT, *n* = 3), sickle cell disease (SCD, *n* = 3), and thalassemia (THL, *n* = 3) (Table 1). In cryptorchid patients, the densities of spermatogonia expressing each of the evaluated markers were comparable to the reference values of the NT group. In contrast, although the density of MAGEA4-positive spermatogonia in SCD patients’ was comparable to the NT group, significantly decreased density of 5mC-positive spermatogonia (*p* < 0.016) was found. In patients diagnosed with THL, the density of UTF1-positive spermatogonia was significantly lower, compared to the NT group (Figure S1).

**Figure 5:**
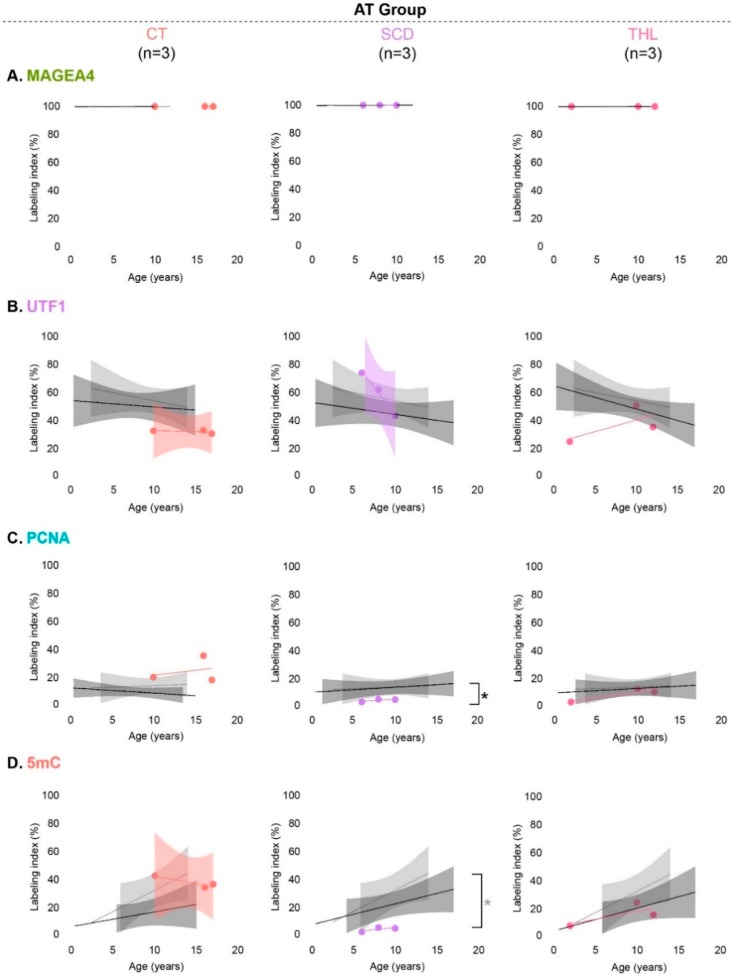
Spermatogonial labeling indices (%) of patients with potentially affected tissues (AT group) particularly due to cryptorchidism (CT), sickle cell disease (SCD), and thalassemia (THL) in comparison with established reference values (NT group) and patients within the AT group without the respective pathology. (**A**) MAGEA4, (**B**) UTF1, (**C**) PCNA, and (**D**) 5mC labeling index. Previously established values throughout development (95% confidence interval) are represented as light-gray area for the NT group and as dark-gray area for patients within the AT group without the respective pathology. Wilcoxon rank-sum test was used to compare labeling indices between groups. Statistically significant differences (* *p* < 0.016) resulting from comparisons with the NT group are represented in light-gray and with patients within the AT group without the respective pathology in black.

Also in these subgroups of patients, labeling indices were determined to assess a potential percentage change in marker-positive spermatogonial subpopulations. Changes were observed only in the SCD patient group. More specifically, these patients had a significantly lower percentage of PCNA-positive spermatogonia compared with remaining samples within the AT group (*n* = 15, *p* < 0.016). Moreover, the SCD patients showed a significantly lower percentage of 5mC-positive spermatogonia (Figure 5D) compared to the reference values (NT group; *p* < 0.016).

## 4. Discussion

In this study, we determined the expression dynamics of spermatogonial subpopulations in the developing human prepubertal testes. By comparison, we have demonstrated that patients who, at the time of fertility preservation, are at risk for germ cell loss due to underlying diseases known to affect testicular function and/or already received potentially gonadotoxic treatment have reduced spermatogonial density while the ratios of spermatogonial subpopulations remain stable.

Due to limited access to healthy pre/pubertal testicular tissue, in our study, the spermatogonial expression patterns during testicular development were established for untreated cancer patients without any preexisting (congenital) risk factors for impaired testicular function. Reassuringly, Stukenborg et al. and Valli-Pulaski et al. recently reported that spermatogonial numbers per tubular cross section of untreated cancer patients were within the confidence interval of spermatogonial reference values previously established in healthy boys [10,15,21].

The numbers of total [21] and undifferentiated (UTF1-positive) [15] spermatogonial populations, as well as proliferative activity [20] are known to vary within age groups. In our patient cohort, the density of MAGEA4-, UTF1- and PCNA-positive spermatogonia showed a trend to increase with age, however without significance. This might be due to the fact that density of marker-expressing spermatogonia was evaluated throughout development as a continuum, to do justice to the interindividual heterogeneity during this period. In contrast, we demonstrate for the first time in human testicular tissues that the density of methylated spermatogonia (5mC-positive) significantly increased with age. The establishment of global DNA methylation in spermatogonia before initiation of spermatogenesis is essential for spermatogonial identity, differentiation potential, and correct transmission of epigenetic information to the next generation [24,34,38]. The importance of correct levels of DNA methylation for germ cell function is demonstrated by data showing the association of infertility with aberrant methylation levels in sperm [47]. Thus, defining the timing of establishment of DNA methylation throughout testicular development is crucial to investigate factors that can lead to disturbances of this process, as disease or exposure to treatments. While the majority of studies in the mouse model have shown perinatal acquisition of germ cells’ DNA methylation [48,49], in non-human primates (marmoset) establishment of methylation was suggested to be a gradual process occurring during postnatal development and completed in adulthood [39]. In agreement with the primate data, our results show that in human, testicular tissue establishment of global DNA methylation of spermatogonia occurs progressively during postnatal and pubertal development. Once established, methylation patterns of spermatogonia are maintained in subsequent differentiation stages [36,50] and are unaltered in adult patients with spermatogenic defects [34].

Accounting for interindividual variability of spermatogonial numbers, analysis of spermatogonial labeling indices were determined revealing that the percentage of MAGEA4-positive spermatogonia remained constant, whereas the percentages of undifferentiated (UTF1-positive), proliferative (PCNA-positive), and methylated (5mC-positive) spermatogonial subpopulations were dynamic during testicular development. The expression of MAGEA4 by most spermatogonia confirmed the diagnostic value of this marker for the detection of spermatogonia in prepubertal testicular tissues. The decreased trend of the spermatogonial labeling index for the undifferentiated subpopulation (UTF1-positive) together with an upward trend of proliferative spermatogonia (PCNA-positive) during testicular development are consistent with the dynamic changes of the seminiferous epithelium at the onset of puberty [23,24]. In our study, UTF1 and PCNA spermatogonial labeling indices of post-pubertal patients reached values identical to those previously described for the adult testis, where UTF1 and proliferating spermatogonia (KI67-positive), respectively, represented 43 ± 3% and 5 ± 2% of the total spermatogonia population [23]. Additionally, the PCNA spermatogonial labeling index we found during adulthood (70 ± 6.1 years) was comparable to the recently reported index for aged man (70.9 ± 6.2 years), which was around 35.4% of A-spermatogonia. Note that the five adult testicular tissues included in this study may not be representative for adult samples in general due to the limited number of samples and the advanced age of the men. This assumption is corroborated by the recent report that increased levels of spermatogonial proliferation throughout adult age can be observed, which might be a compensatory mechanism to maintain spermatogenesis [30].

Besides providing new insights to the current knowledge of spermatogonial subpopulation dynamics during human testicular development, our established reference expression patterns allowed the evaluation of the effect of treatment and/or diseases on spermatogonial composition. The patients included in the AT group showed a reduced density of UTF1-, PCNA-, and 5mC-positive spermatogonia. The constant density of MAGEA4-positive spermatogonia, but reduced densities of UTF1- and PCNA-positive spermatogonia throughout prepubertal age suggest that undifferentiated and proliferative spermatogonia are vulnerable to gonadotoxic treatment and/or diseases. Previous reports also showed no alterations in MAGEA4 spermatogonial density in prepubertal patients who received chemotherapy [4,5]. Further studies demonstrated that spermatogonial numbers do not differ between untreated cancer patients and patients receiving non-alkylating agents as treatment regime. In contrast, a significant spermatogonial depletion was observed in boys receiving alkylating therapies [9,10]. Recently, Valli-Pulaski et al. showed in a cohort of 137 patients no impact of previous exposure to either alkylating or non-alkylating chemotherapy on the number of UTF1-positive spermatogonia compared with patients prior to treatment [15]. This contradicting outcome may be due to the different approaches for spermatogonial quantification between studies. Although Valli-Pulaski et al. determined spermatogonial numbers per testicular tubular cross section, we quantified spermatogonia density within the total tissue section. We consider it highly likely that the spermatogonial density will be an essential factor for the outcome of experimental approaches aiming for fertility restoration [5]. This assumption is based on data reporting low efficiencies of spermatid production using in vitro spermatogenesis or recolonization of the testes following germ cell transplantation. Importantly, these data were derived employing intact testicular tissues from model organisms as starting material [51,52,53,54,55,56,57]. It is therefore likely that the chances for the successful use of immature human testicular tissues for future fertility restoration approaches will depend on the number and quality of spermatogonia present in the starting material.

To date, evaluation of spermatogonial methylation patterns in patients at risk of germ cell loss has never been considered. Our results show that disease and/or treatment (AT group) can impact establishment of spermatogonial methylation which might reflect delayed development and have consequences for initiation, completion, and maintenance of spermatogenesis. 

It is well recognized that cryptorchidism is associated with a reduction of germ cell numbers, possibly depending on the age at orchidopexy [58,59,60,61]. Surprisingly, spermatogonial densities of cryptorchidic patients included in our cohort were comparable to the established reference values (NT group) for all evaluated markers. This data should however be interpreted with caution considering the low number of patients with cryptorchidism that were included in our study. Moreover, an approach providing higher resolution of morphological details, such as electron microscopy, may be required to gain insight into the potential changes of spermatogonial subtypes A_dark_, A_pale_, and B [62,63].

Testes of prepubertal boys treated with hydroxyurea for sickle cell disease were described to be depleted of spermatogonia [10,15]. Although spermatogonia expressing MAGEA4 were observed in treated sickle cell disease patients within our cohort with a density comparable to the NT reference group, a reduction of the 5mC spermatogonial subpopulation suggests a possible developmental arrest. Although this was corroborated by an increase of the undifferentiated UTF1-positive spermatogonia, this was not statistically significant, possibly due to the low number of patients. Although follow-up studies need to be performed, the altered spermatogonial methylation might be a possible reason for the previously reported spermatogonial depletion after hydroxyurea treatment since correct establishment of methylation is necessary for spermatogonial integrity and differentiation. Interestingly, in previous studies hydroxyurea treatment was also correlated with hypomethylation (specifically of the γ-globin gene promoter), albeit without statistical significance [64].

## 5. Conclusions

Our study provides crucial information on spermatogonial subpopulations of prepubertal boys included in fertility preservation programs. Despite the reduction of the spermatogonial density in the AT group compared to normal testicular development, the spermatogonial composition (labeling indices) remained unchanged. This result is of importance since the presence of unaltered spermatogonial subpopulations is crucial for fertility preservation and restoration. Interestingly, significantly lower density and percentage of 5mC-positive spermatogonia in patients with sickle cell disease indicate an arrested germ cell development. As this is a growing patient group in fertility preservation programs, this finding necessitates further analysis in follow-up studies to clarify the potential use of these cryopreserved tissue samples.

## Figures and Tables

**Table 1:** Baseline characteristics of the patient cohort. According to diagnosis, treatment received and occurrence of any testicular pathology, patients were divided into two groups: NT: untreated cancer patients without any preexisting (congenital) risk factors for impaired testicular function; AT: patients with potentially affected tissues by disease or treatment. The average age of the patients in NT and AT group is not significantly different (9.1 and 9.5 years, respectively).

Group	Patient Number	Age (Years)	Testis Volume (mL)	Diagnosis (Treatment before Testis Biopsy)
**NT**	NT1	2.5	1.5	Ependymoma
	NT2 *	3	0.33	Acute myeloid leukemia
	NT3	5	1	Hodgkin’s lymphoma
	NT4	6	1	Hodgkin’s lymphoma
	NT5	7	0.83	Rhabdomyosarcoma
	NT6	8	0.40	Ewing sarcoma
	NT7	9	1.30	Intracranial germ cell tumor
	NT8	10.5	1.80	Rhabdomyosarcoma
	NT9	11	1	Hodgkin’s lymphoma
	NT10	11	2	Osteosarcoma
	NT11	13	1	Hodgkin’s lymphoma
	NT12	14	8 ^#^	Sarcoma
	NT13	14	19	Lymphoma
	NT14	14	9	Non-Hodgkin’s lymphoma
**AT**	AT1	5 months	-	Immunodeficiency (chemotherapy)
	AT2	2	0.3	Thalassemia
	AT3	3	8	Testicular tumor, pubertas praecox
	AT4	4	0.4	Immunodeficiency
	AT5	6	-	Leukemia (allogeneic bone marrow transplantation)
	AT6	6	1	Sickle cell disease (hydroxyurea)
	AT7	7	1	Myelodysplastic syndrome
	AT8 *	7	1	Sickle cell disease, cryptorchid testes
	AT9	8	0.3	Sickle cell disease (hydroxyurea)
	AT10	9	1	Myelodysplastic syndrome
	AT11	10	1	Thalassemia major
	AT12	10	1	Hodgkin’s lymphoma, cryptorchid testes
	AT13	10	2	Sickle cell disease (hydroxyurea)
	AT14	12	1.4	Myelodysplastic syndrome
	AT15 *	12	-	Cryptorchid testes, hypogonadism
	AT16	12	1	Thalassemia major
	AT17	13	8	Leukemia (chemotherapy)
	AT18 *	14	6	Cryptorchid testes
	AT19	15	6	Lymphoma (chemotherapy)
	AT20	16	5	Leukemia, cryptorchid testes (chemotherapies)
	AT21 *	16	3	Testicular tumor, cryptorchid testes
	AT22	17	3	Cryptorchid testes

* Patients with low spermatogonial counts and a patient with germ cell neoplasia in situ (AT21) were excluded from further analysis. Testicular volumes were determined by palpation using a Prader orchidometer and additionally confirmed by ultrasonography. ^#^ Indicates one patient for whom testicular volume was only determined by Prader orchidometer.

## Supplementary Materials

The following are available online at https://www.mdpi.com/2077-0383/9/1/224/s1, Figure S1: Spermatogonial marker-specific density in patients with potentially affected testicular tissue (AT group) particularly due to cryptorchidism (CT), sickle cell disease (SCD), and thalassemia (THL) in comparison with established reference values (NT group) and patients within the AT group without the respective pathology. Table S1: Primary antibodies and immunohistochemistry protocol specifications. Table S2: Total number of evaluated cells and density of spermatogonia (Spg) evaluated for each patient (NT and AT groups) and adult controls (Ctrl).
