# Supplementary material for: Development and Disease-Dependent Dynamics of Spermatogonial Subpopulations in Human Testicular Tissues
Source: J Clin Med. 2020 Jan 14;9(1):224. doi: 10.3390/jcm9010224 (PMC7019285; doi:10.3390/jcm9010224)
Supplement: Supplementary file 1 [file jcm-09-00224-s001.pdf]

Supplementary Materials

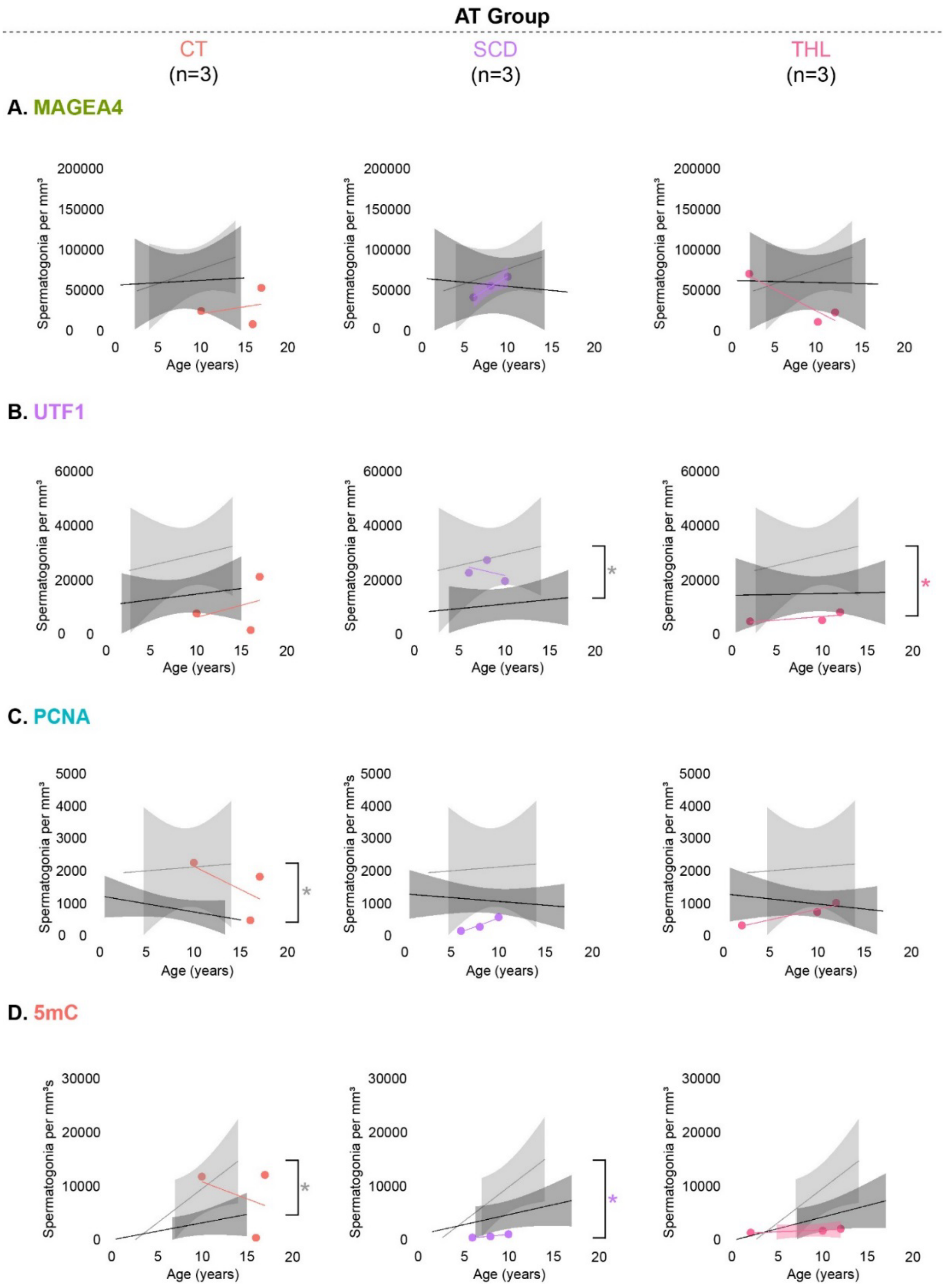

Figure S1. Spermatogonial marker-specific density in patients with potentially affected testicular tissue (AT group) particularly due to cryptorchidism (CT), sickle cell disease (SCD) and thalassemia (THL) in comparison with established reference values (NT group) and patients within the AT group without the respective pathology. (**A**) MAGEA4, (**B**) UTF1, (**C**) PCNA, and (**D**) 5mC spermatogonial density. Previously established values throughout development (95% confidence interval) are represented as light-gray area for the NT group and as dark-gray area for patients within the AT group without the respective pathology. Wilcoxon rank-sum test was used to compare spermatogonial density between groups. Statistically significant differences (*p<0.016) resulting from comparisons between the NT group and AT patients without the respective disease are represented

in light-gray while comparisons between the NT group and AT patients with the respective disease are colored represented.

Table S1. Primary antibodies and immunohistochemistry protocol specifications.

Antibody	Germ cell type	Localization	Conc.	Catalog number	Reference
**MAGEA4**	Spermatogonia, primary spermatocytes	Cytoplasm	1:20	Provided by Prof. Spagnoli	Aubry *et al.*, 2001 Heckmann *et al.*, 2018
**UTF1**	Gonocytes, undifferentiated spermatogonia	Nucleus	1:50	MAB4377	Kristensen *et al.*, 2008 von Kopylow *et al.*, 2010
**PCNA**	Spermatogonia, spermatocytes (proliferation)	Nucleus	1:200	ab92552	Steger *et al.*, 1998
**5mC**	Spermatogonia to spermatids (methylation)	Nucleus	1:200	C15200081-100	Adiga *et al.*, 2011 Nettersheim *et al.*, 2013

Abbreviations: MAGE A4, Melanoma-associated antigen 4; UTF1, Undifferentiated embryonic cell transcription factor 1; PCNA, Proliferating cell nuclear antigen; 5mC, 5-methylcytosine.

Table S2. Total number of evaluated cells and density of spermatogonia (Spg) evaluated for each patient (NT and AT groups) and adult controls (Ctrl).

		MAGEA4			UTF1			PCNA			5mC		
		Total no.	Spg. per mm^3		Total no.	Spg. per mm^3		Total no.	Spg. per mm^3		Total no.	Spg. per mm^3	
**Patients**	**Age (years)**	**Spg.**	**Positive**	**Negative**	**Spg.**	**Positive**	**Negative**	**Spg.**	**Positive**	**Negative**	**Spg.**	**Positive**	**Negative**
**NT1**	2,5	353	79436,3	680,9	203	20169,4	24823,9	327	2820,9	43300,3	246	4938,8	29774,1
***NT2**	3	7	2261,0	0,0	7	1934,3	773,7	8	0,0	3843,2	20	2125,3	8501,4
**NT3**	5	233	25653,3	0,0	254	19212,0	10725,7	67	865,3	3275,6	179	214,4	12539,9
**NT4**	6	780	37776,6	0,0	538	19509,8	9974,1	511	147,6	18706,0	116	7210,3	9030,4
**NT5**	7	173	40269,1	0,0	128	19606,0	7378,6	94	4058,9	17137,6	184	4742,9	24346,8
**NT6**	8	121	72975,5	0,0	50	10617,0	20609,5	78	11292,5	30651,0	35	5853,8	35122,9
**NT7**	9	128	93242,0	0,0	127	52455,8	41373,6	44	1383,7	29058,3	44	10767,4	20817,0
**NT8**	11	576	160637,7	0,0	415	57859,9	58139,4	371	4686,4	64860,4	377	4869,1	74941,5
**NT9**	11	521	68128,0	0,0	508	48462,3	18255,5	199	980,6	20701,4	146	8694,3	6785,8
**NT10**	11	538	62330,0	0,0	475	38017,1	15567,8	129	2138,4	8895,8	122	3941,2	9796,7
**NT11**	13	1994	51182,5	0,0	1958	26845,3	24286,1	542	78,1	10502,5	676	1357,6	12751,5
**NT12**	14	2705	51839,4	57,6	2250	12571,9	38765,2	2804	8565,2	26753,8	4226	36966,7	14934,0
**NT13**	14	1337	44654,1	0,0	1199	17488,7	26563,7	390	1268,0	8428,4	2526	29338,0	16869,1
**NT14**	14	3490	181224,1	0,0	2753	75334,7	69394,2	959	4429,2	22118,3	2416	19781,7	46262,2
**AT1**	5 mo	352	26915,8	231,4	244	11959,2	6627,0	274	2459,3	8771,6	146	0,0	7268,6
**AT2**	2	854	69777,9	0,0	241	4510,4	14560,0	319	292,8	16663,4	469	1234,4	18707,1
**AT3**	3	641	37118,3	0,0	657	16826,9	21426,7	201	1278,6	6045,9	311	7586,0	7274,9
**AT4**	4	3883	183558,6	0,0	3333	104508,3	52962,9	436	494,2	10004,8	907	937,1	36817,1
**AT5**	6	424	21038,5	0,0	321	7748,4	6383,6	414	857,3	12033,5	273	639,2	9332,3
**AT6**	6	522	40898,0	0,0	432	22482,7	8059,8	56	123,1	6709,7	111	85,0	9305,7
**AT7**	7	490	27016,2	0,0	359	9710,3	10916,9	237	1262,0	13692,2	242	31,2	7530,4
***AT8**	7	30	1397,2	0,0	0	np	np	5	40,9	163,5	49	1389,7	2866,3
**AT9**	8	282	55021,8	0,0	233	27182,5	17107,8	79	248,1	6243,7	75	285,3	6800,1
**AT10**	9	2148	151266,1	0,0	4344	86275,7	206293,9	137	1253,1	11952,7	480	5290,9	32056,4
**AT11**	10	94	10475,0	0,0	88	4946,2	4946,2	122	704,2	5633,9	139	1602,3	5332,5
**AT12**	10	701	24041,0	0,0	677	7471,3	16275,4	403	2227,0	9581,9	890	11678,1	16374,6
**AT13**	10	92	66139,7	0,0	80	19386,0	26228,1	128	547,3	14960,9	207	707,2	20155,6
**AT14**	12	401	17172,4	0,0	246	7960,3	2682,3	319	405,6	8820,8	727	2345,3	22012,6
***AT15**	12	120	4122,0	0,0	113	1370,9	2407,4	3	0,0	59,2	2	0,0	45,9
**AT16**	12	265	22238,5	0,0	286	7919,2	15191,8	191	986,6	9781,1	242	1942,9	11657,2
**AT17**	13	154	14226,0	0,0	98	2806,1	6360,6	64	893,4	4824,4	55	645,6	3792,8
***AT18**	14	0	np	np	0	np	np	0	np	np	0	np	np
**AT19**	15	3074	165051,9	0,0	2336	35988,4	32864,1	1040	471,7	19152,2	1251	13525,1	15722,8
**AT20**	16	179	7754,3	0,0	91	1274,3	2724,3	63	452,2	862,4	52	377,4	765,9
**#AT21**	16	436	7984,6	4566,9	283	0,0	8474,3	199	1639,3	1926,0	300	0,0	5604,3
**AT22**	17	1075	52411,7	0,0	1387	21021,3	50266,1	247	1793,2	8879,5	511	11983,0	21814,3
**Ctrl1**	62	9521	122157,0	0,0	2964	50689,1	61936,4	1351	8001,4	23050,1	2326	41365,8	30057,0
**Ctrl2**	65	9569	119612,7	0,0	2987	51390,7	66056,4	2220	14027,7	20587,7	2214	70455,0	5669,2
**Ctrl3**	67	14267	139706,5	0,0	4652	83539,9	44089,4	4258	11859,3	32084,1	3401	62989,4	28702,5
**Ctrl4**	68	11322	128400,8	0,0	3035	51015,1	38682,9	1602	2021,6	12150,4	2159	69032,8	19739,2
**Ctrl5**	74	8101	171070,1	0,0	2907	100940,3	63996,3	1583	14864,7	26499,1	2093	30148,9	30890,2
**Ctrl6**	75	11090	141704,4	0,0	3274	46695,5	62910,8	1818	16313,4	27609,4	2630	85347,3	29803,1
**Ctrl7**	79	8663	118108,1	0,0	2553	41255,4	59587,4	1010	4374,5	13927,4	2347	35176,8	32053,1

Heat-map showing individual values for positive and negative spermatogonia. Color scale from red to blue respectively indicates relatively high to low values of spermatogonial density. * Patients with low spermatogonial counts and a # patient with germ cell neoplasia *in situ* were excluded from further analysis; np- not present.
